# Preliminary data on arsenic and trace metals concentrations in wetlands around artisanal and industrial mining areas (Cote d’Ivoire, West Africa)

**DOI:** 10.1016/j.dib.2018.04.105

**Published:** 2018-05-01

**Authors:** Kakou Charles Kinimo, Koffi marcellin Yao, Stephane Marcotte, N.’Guessan Louis Berenger Kouassi, Albert Trokourey

**Affiliations:** a Laboratoire de Chimie Physique, Université Félix Houphouët Boigny, 22 BP 582 Abidjan, Côte d’Ivoire; b Centre de Recherches Océanologiques (CRO), 29, rue des pêcheurs, BP V18 Abidjan, Côte d’Ivoire; c Normandie Université, COBRA, UMR CNRS 6014 et FR 3038, Université de Rouen et INSA de Rouen, 1 rue Tesnière, 76821 Mont Saint-Aignan Cedex, France; d Université Peleforo Gon Coulibaly, UFR des Sciences Biologiques, BP 1328 Korhogo, Côte d’Ivoire

## Abstract

This data article is issued from the research article ‘’Distribution trends and ecological risks of arsenic and trace metals in wetland sediments around gold mining activities in central-southern and southeastern Côte d’Ivoire’’ [1]. It presents arsenic and trace metal Cd, Pb, Cu, Zn, Cr, Co, Fe, Al, Mn, and Ni loadings in surface sediments collected from industrial mining, artisanal and small scale mining, and non-mining areas (West Africa). Are also provided, hierarchical dendrograms and factor loadings derived from the Hierarchical Clustering Analysis (HCA) and the principal component analysis (PCA), respectively. Data ranged from <LD-561 µg/g for As, 0.10–2.70 µg/g for Cd, 1.10–16.9 µg/g for Pb, 2.00–71.8 µg/g for Cu, 5.60–116 µg/g for Zn, 16.3–439 µg/g for Cr, 0.70–46.4 µg/g for Co, 11.9–175 mg/g for Fe, 7.49–93.3 mg/g for Al, 4.30–6442 µg/g for Mn, and 3.10–68.6 µg/g for Ni. These data are relevant for future research and sediment quality policy making at a regional level.

**Specifications table**

**Table:** 

Subject area	*Chemistry, Biology*
More specific subject area	*Biogeochemical cycle of metal(loid)s*
Type of data	*Table, figure*
How data was acquired	*Inductively coupled plasma-optical emission spectrometer (ICP OES Icap 6200, Thermo Fisher, Cambridge, UK)*
Data format	*Raw and analyzed*
Experimental factors	*A total of 45 surface sediment samples were air-dried, ground, sieved and total digested* using a microwave-assisted digestion system (Milestone Ethos 1 microwave, Shelton, US).
Experimental features	*Measuring TOC and total metal concentrations; performing Principal Component Analysis (PCA) and Hierarchical Clustering Analysis (HCA); comparing data with literature.*
Data source location	*Afema* (05.41 °N, 02.92 °W)*, Agbaou and Bonikro (06.38 °N, 05.21 °W), central-southern and south-eastern Côte d'Ivoire, West Africa.*
Data accessibility	*Data is with this article*

**Value of the data**

• Intensive industrial and small scale gold mining occur in West Africa
• Little information available on trace metals in wetland sediments around gold mining activities
• First As and Cd, Pb, Cu, Zn, Cr, Co, Fe, Al, Mn, and Ni data are provided in Côte d'Ivoire
• Data will help develop future research on metal(loid)s fate in mining and cash crops farming agricultural areas within West Africa

## 1 Data

Arsenic and trace metals total concentrations in sediment samples collected in industrial, artisanal and non-mining stations from Afema, Agbaou and Bonikro gold mine sites were shown in Supplementary Table 1. Fig. 2 show hierarchical dendograms performed with TOC, arsenic and trace metals concentrations in sediments from the three studied gold mine sites. Comparison of average concentrations of arsenic and trace metals with data from similar areas in the world is presented in Table 1. Table 2 shows factor loadings of TOC, arsenic and trace metals concentrations from PCA analysis for Afema, Agbaou and Bonikro gold mining areas.

**Table 1:** Comparison of average (±Standard deviation) concentrations of arsenic and trace metals with data from similar areas in the world.

**Location**	***n***	**As**	**Cd**		**Co**	**Cr**	**Cu**	**Mn**	**Ni**	**Pb**	**Zn**	References
Afema	15	102±177	0.60±0.46		11.0±5.30	86.7±56.4	16.2±6.20	212±171.9	23.5±14.6	**4.70±2.70**	**39.7±17.5**	This study
Agbaou	15	6.2±12.5	0.60±0.25		29.4±14.1	131±42.1	30.9±15.9	2484±2696	40.9±19.9	**5.80±2.20**	**38.8±10.7**	This study
Bonikro	15	14.2±27.6	0.81±0.61		15.8±6.2	145±139	27.3±18.4	394±260	22.5±12.7	**9.10±4.60**	**47.2±28.7**	This study
**Africa**												
Nigeria	10	1.7±0.4	0.43±0.1		0.85±1.40	4.40±13.0	–	–	–	6.00±11.0	9.60±18.0	[7]
Nigeria	13	nd^a^	nd		–	–	–	–	13.6±11.8	15.2±6.7	49.8±32.0	[8]
Ghana	20	6.83±2.42	0.19±0.1		–	16.8±2.47	17.3±17.5	–	47.8±13.0	20.0±3.11	61.9±16.5	[9]
Ghana	60	3.80±3.40	0.03±0.0		1.30±0.70	16.0±10.0	16.0±3.22	446±108	2.20±1.00	4.80±3.50	37.0±24.0	[10]
Senegal	6	20.2±20.5	–		27.6±10.5	618±533	5.10±1.90		55.3±39.8	15.5±6.30	236±367	[11]
Burkina Faso	16	40.3±80.8	nd		19.5±7.10	93.1±79.3	38.5±24.7	–	32.0±30.4	10.5±11.9	30.8±30.3	[12]
Congo	14	63.9±34.1	nd		3774±3948	93.3±42.5	69.0±47.5	–	55.4±28.9	1545±2607	1415±1831	[13]
**Other countries**												
Bolivia	24	50.0±5.50		–	–	–	–	–	–	–	–	[14]
Canada	21	7.10±1.90		0.6±0.0	–	–	–	–	–	–	100±13.0	[15]
China	407	11.6±7.10		0.24±0.2	–	72.1±33.9	53.5±69.2	307.2±159.5		47.0±29.8	87.9±55.7	[16]
India	47	2.40±1.40		–	21.5±14.9	499±916	24.0±22.0		168±286	11.3±11.2	98.5±86.5	[17]
India	12	17,442± 33,148			28.8±2.40	95.2±10.3		1715±1941	67.8±14.5	19.8±11.6	92.5±31.7	[18]
France	27	1028±1289		27±42	–	–	–	649±894	–	–	1955±1754	[19]
Mexico	20	2898±3481		67.6±83.2	–	–	511±603	–	–	27,079± 33,900	14,284±171	[20]
Columbia	17	–							55.6±10.8	3.81±1.62	142.2±19.7	[21]
UCC		**2.0**		**0.102**	**11.6**	**35**	**14.3**	**527**	**18.6**	**17**	**52**	[22]

a nd: non detected; ‘’-‘’ not measured.

**Table 2:** Sorted rotated factor loadings (Varimax normalized) of arsenic, trace metals and total organic carbon (TOC) in the three principal factors derived from the principal component analysis (PCA) for Afema, Agbaou and Bonikro gold mining areas.

Trace metals	Afema			Agbaou			Bonikro		
	Fact.1	Fact.2	Fact.3	Fact.1	Fact.2	Fact.3	Fact.1	Fact.2	Fact.3
Al	**−0.76**	**−0.59**	0.03	−0.02	−0.46	**0.84**	**0.79**	−0.17	0.44
As	**0.90**	−0.29	0.00	**−0.60**	**0.62**	0.36	**0.73**	−0.02	−0.36
Cd	0.22	−0.25	**−0.92**	0.26	**−0.79**	0.23	**0.94**	−0.03	−0.08
Co	**−0.63**	**0.52**	**−0.52**	**0.98**	0.06	0.16	0.14	**0.81**	0.11
Cr	**−0.61**	**0.63**	0.14	**0.72**	−0.50	0.33	0.22	0.11	**−0.69**
Cu	−0.12	**−0.94**	−0.06	−0.20	**0.78**	**0.52**	**0.87**	−0.20	0.23
Fe	0.23	−0.30	**−0.88**	−0.41	0.07	**0.86**	**0.97**	−0.03	−0.18
Mn	−0.19	**0.63**	**−0.57**	**0.87**	0.43	−0.12	−0.25	**0.90**	0.11
Ni	**−0.73**	**−0.58**	0.00	**0.92**	0.32	0.18	**0.63**	0.17	**0.66**
Pb	**0.85**	−0.22	−0.08	**−0.68**	−0.51	0.31	0.40	0.02	**−0.76**
Zn	**−0.86**	0.00	−0.17	**0.80**	0.04	**0.49**	−0.07	**−0.64**	0.31
TOC	**−0.59**	**−0.71**	0.01	0.02	**−0.68**	−0.24	0.38	**0.78**	0.14
Eigen value	4.66	3.42	2.27	4.81	3.10	2.46	4.59	2.61	2.04
Total variance (%)	38.84	28.49	18.92	40.07	25.80	20.51	38.28	21.79	16.97
Cumulative Variance (%)	38.84	67.34	86.26	40.07	65.87	86.38	38.28	60.06	77.03

## 2 Experimental design, materials and methods

### 2.1 Study area

The study was carried out in wetlands around Agbaou, Bonikro (06.38 °N, 05.21 °W), and Afema (05.41 °N, 02.92 °W) gold mines. The two formers are located in central-southern Côte d’Ivoire (Fig. 2). The geology of Bonikro and Agbaou deposits is dominated by a granodiorite intrusion (felsic) into mafic volcanics (basalts) of the upper Birimian Series that have been metamorphosed to mid greenschist facies, and sedimentary rocks, with a strike length of 1000 m and a width of up to 300 m [2]. The geology of Afema is also characterized by lithology of Birimian, with mainly metalovolcanic and a metasedimentary rock assemblage which can be followed up to the adjacent Ghanaian territory [3].

### 2.2 Sampling and chemical analysis

All sampling devices were cleaned by rinsing with pure water and kept in 0.1 M HNO_3_ (68%, Fischer Scientific) for several days before sampling.

**Fig. 1:**
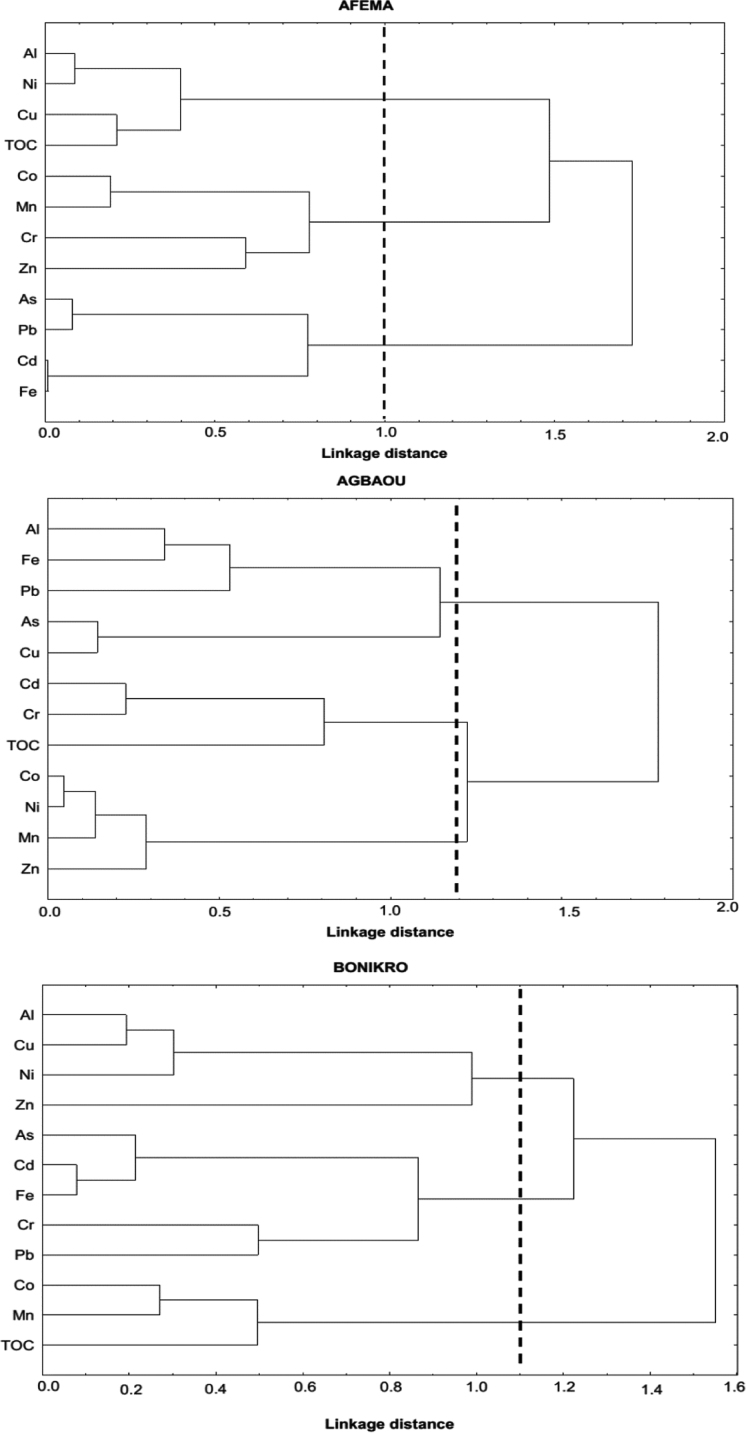
Hierarchical dendrograms for TOC, arsenic and trace metal concentrations in sediments collected from Afema, Agbaou, and Bonikro mining areas.

A total of 45 surface sediment samples (0–5 cm) were collected from Mars to November 2015 around industrial mining, artisanal mining, and non-mining areas (Fig. 1). Each sample (300 g) was made of five subsamples collected using a Van Veen stainless steel grab (with an area of 0.02 m^2^) [4]. Samples were then put into ice bags and transported to the laboratory, air-dried at room temperature, ground with an agate mortar to pass through a 63 µm sieve, and stored in polyethylene zip-type bags and shipped to Laboratoire de Chimie Organique Bioorganique Réactivité et Analyse (COBRA), Université de Rouen, France for further analysis.

**Fig. 2:**
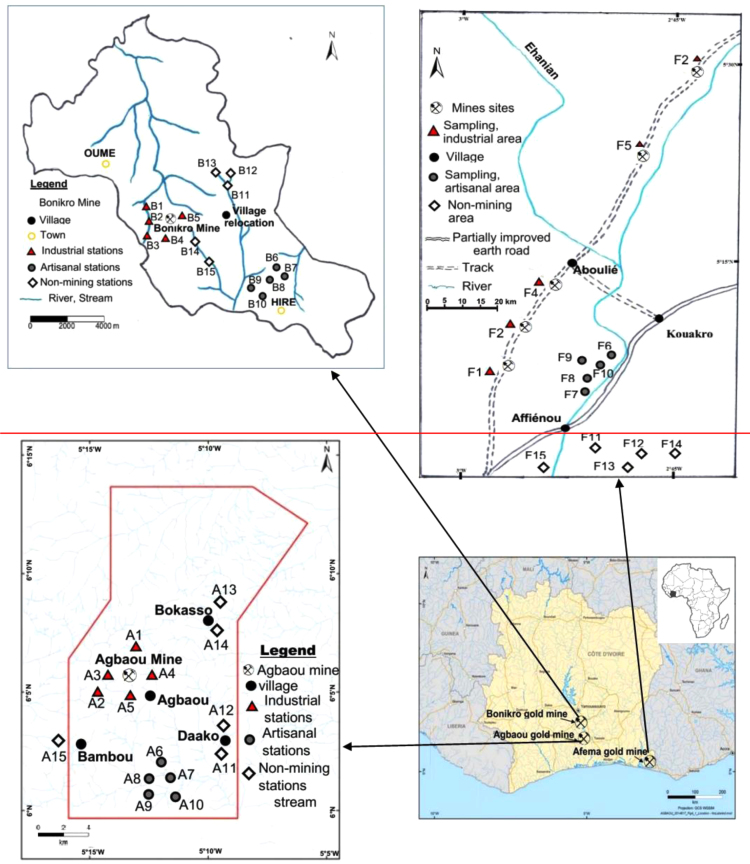
Location of sampling areas and stations.

Sediment samples were digested using a microwave-assisted digestion system (Milestone Ethos 1 microwave, Shelton, US), following Method 3051 A [5]. About 0.5 g of homogenized sediments were first left react with a mixture of 3 mL 68% HNO_3_ and 9 mL 37% HCl (trace metal grade, Fisher Scientific) in loosely capped Teflon reactors for 30 min at room temperature, in a fume hood, to avoid an overpressure during the heating step (USEPA [5]). Then, the digestion was performed under high power at programmed temperatures and time intervals (Table 3). After cooling, the solutions were diluted to 50 mL with ultrapure 2% HNO_3_ in Teflon tubes and centrifuged at 4000 rpm for 5 min prior analysis of the supernatant. Duplicate blanks were prepared and analyzed with each batch of digested samples [1].

**Table 3:** Operating conditions for microwave digestion systems (Milestone Ethos 1 microwave, Shelton, US).

**Time**	**Temperature (°C)**	**Power (W)**
0	25	0
10	150	800
15	150	800
20	165	800
25	165	800
30	180	800

Trace metals (Cd, Pb, Cu, Zn, Cr, Co, Fe, Al, Mn, and Ni) and arsenic were measured using an inductively coupled plasma-optical emission spectrometer (ICP OES Icap 6200, Thermo Fisher, Cambridge, UK). Three replicates of each sample analyzed presented an error that was within 6%. Accuracy of the analytical procedures were evaluated through the analysis of the certified reference material CRM CNS 301-04-050 (Sigma-Aldrich; Missouri, U.S.A) for freshwater sediment. The measured concentrations fell within the range of certified values and the recoveries varied between 85% and 110%.

Total organic carbon (TOC) was determined by loss on ignition (in percentage) of 1.0 g of dried sediments in an oven at 550 °C for 4 h [6]. The precision of three triplicate analyses of each sample fell within error ranges of 5–10%.

### 2.3 Statistical analyses

Principal Component Analysis (PCA) and Hierarchical Clustering Analysis (HCA) were performed using Statistica 7.1 Software. Averages and standards deviations were calculated using Microsoft Office Excel 2013.
