# Supplementary material for: Preliminary data on arsenic and trace metals concentrations in wetlands around artisanal and industrial mining areas (Cote d’Ivoire, West Africa)
Source: Data Brief. 2018 May 1;18:1987–94. doi: 10.1016/j.dib.2018.04.105 (PMC5998745; doi:10.1016/j.dib.2018.04.105)
Supplement: Supplementary file 1 — Supplementary material [file mmc1.docx]

**Conflict of Interest**

The authors declare no conflict of interests regarding this data article.
