# Supplementary material for: Preliminary data on arsenic and trace metals concentrations in wetlands around artisanal and industrial mining areas (Cote d’Ivoire, West Africa)
Source: Data Brief. 2018 May 1;18:1987–94. doi: 10.1016/j.dib.2018.04.105 (PMC5998745; doi:10.1016/j.dib.2018.04.105)
Supplement: Supplementary file 2 — Supplementary material [file mmc2.docx]

**Table 1**: Average arsenic and trace metal concentrations (µg g^-1^), and Standard deviation (SD) in samples of surface sediments from Afema, Agbaou, Bonikro gold mining areas

| **Sampling sites** | **Afema gold mine site** | | | | | | | | | | |
| --- | --- | --- | --- | --- | --- | --- | --- | --- | --- | --- | --- |
| **Industrial stations** |  |  |  |  |  |  |  |  |  |  |  |
|  | **Al** | **As** | **Cd** | **Co** | **Cr** | **Cu** | **Fe** | **Mn** | **Ni** | **Pb** | **Zn** |
| F1 | 11511 | 561 | 0.20 | 0.70 | 16.3 | 19.0 | 15829 | 104 | 3.10 | 12.5 | 5.6 |
| F2 | 12293 | 471 | 0.80 | 4.50 | 60.2 | 12.9 | 52021 | 64.7 | 13.0 | 8.20 | 22.7 |
| F3 | 41564 | 33.3 | 0.40 | 8.50 | 43.5 | 17.4 | 22530 | 106 | 14.9 | 3.80 | 44.4 |
| F4 | 39015 | 245 | 2.10 | 12.2 | 78.2 | 22.4 | 13496 | 309 | 29.5 | 7.00 | 29.1 |
| F5 | 23786 | 151 | 0.90 | 4.90 | 47.5 | 23.9 | 61271 | 118 | 14.2 | 4.90 | 16.3 |
| **Average** | **25634** | **294** | **0.88** | **6.16** | **49.1** | **19.1** | **57320** | **140** | **14.9** | **7.28** | **23.6** |
| **SD** | **12757** | **195** | **0.66** | **3.90** | **20.4** | **3.88** | **42.431** | **86** | **8.44** | **3.03** | **13.0** |
| **Artisanal stations** | | | | | | | | | | | |
| F6 | 56512 | 13.6 | 0.90 | 22.2 | 111 | 19.7 | 52043 | 487 | 33.9 | 4.90 | 79.1 |
| F7 | 60483 | 3.40 | 0.60 | 11.5 | 112 | 15.9 | 43264 | 157 | 29.1 | 4.90 | 46.9 |
| F8 | 53673 | 4.10 | 0.50 | 10.3 | 103 | 14.7 | 34829 | 159 | 27.9 | 1.10 | 48.9 |
| F9 | 71033 | 2.30 | 0.10 | 8.00 | 214 | 23.5 | 13141 | 44.2 | 57.3 | 2.40 | 43.8 |
| F10 | 75860 | 2.10 | 0.30 | 9.20 | 216 | 27.0 | 22543 | 45.8 | 52.3 | 2.80 | 58.4 |
| **Average** | **63512** | **5.10** | **0.48** | **12.2** | **151** | **20.2** | **33164** | **179** | **40.1** | **3.22** | **55.4** |
| **SD** | **8533** | **4.31** | **0.27** | **5.11** | **52.1** | **4.61** | **13964** | **162** | **12.3** | **1.48** | **12.8** |
| **Non-mining stations** | | | | | | | | | | | |
| F11 | 24925 | 10.5 | 0.40 | 11.2 | 56.1 | 8.20 | 26087 | 146 | 15.3 | 4.30 | 33.9 |
| F12 | 20314 | 7.00 | 0.40 | 17.7 | 49.6 | 6.90 | 24421 | 632 | 12.5 | 3.30 | 29.7 |
| F13 | 22547 | 4.70 | 0.60 | 16.0 | 73.7 | 2.00 | 31431 | 240 | 20.1 | 3.60 | 56.7 |
| F14 | 22501 | 7.10 | 0.37 | 14.6 | 58.7 | 8.49 | 27302 | 340 | 14.9 | 3.65 | 38.4 |
| F15 | 22496 | 6.87 | 0.40 | 14.9 | 59.4 | 9.86 | 27295 | 329 | 15.0 | 2.97 | 41.0 |
| **Average** | **22557** | **7.22** | **0.43** | **14.7** | **59.5** | **9.09** | **27307** | **338** | **15.6** | **3.56** | **39.9** |
| **SD** | **1883** | **2.39** | **0.10** | **2.76** | **10.2** | **2.16** | **2990** | **210** | **3.14** | **0.43** | **11.9** |
| **Sampling site** | **Agbaou gold mine site** | | | | | | | | | |  |
| **Industrial stations** | **Al** | **As** | **Cd** | **Co** | **Cr** | **Cu** | **Fe** | **Mn** | **Ni** | **Pb** | **Zn** |
| A1 | 47430 | 48.3 | 0.30 | 10.30 | 174 | 71.8 | 86721 | 94.9 | 25.5 | 5.80 | 26.5 |
| A2 | 17132 | ˂LD | 0.40 | 7.00 | 40.3 | 5.00 | 11910 | 117 | 8.00 | 6.60 | 13.9 |
| A3 | 61109 | ˂LD | 0.40 | 14.0 | 217 | 24.2 | 104414 | 314 | 26.1 | 10.7 | 46.6 |
| A4 | 11861 | ˂LD | 0.60 | 10.4 | 50.3 | 17.3 | 12901 | 168 | 13.0 | 5.50 | 21.3 |
| A5 | 34147 | 23.9 | 0.30 | 13.7 | 166 | 60.7 | 106463 | 142 | 23.8 | 7.70 | 30.1 |
| **Average** | **34336** | **36.1** | **0.40** | **11.1** | **130** | **35.8** | **64482** | **168** | **19.3** | **7.26** | **27.7** |
| **SD** | **18381** | **12.2** | **0.11** | **2.57** | **71.0** | **25.8** | **43072** | **77.3** | **7.38** | **1.88** | **10.9** |
| **Artisanal stations** | | | | | | | | | | |  |
| A6 | 28287 | ˂LD | 0.40 | 45.3 | 122 | 34.2 | 40534 | 6442 | 66.3 | 3.90 | 49.0 |
| A7 | 26478 | ˂LD | 0.40 | 42.6 | 114 | 32.0 | 38202 | 6083 | 62.5 | 3.90 | 46.4 |
| A8 | 35881 | ˂LD | 0.60 | 46.1 | 125 | 35.2 | 41402 | 6249 | 68.5 | 2.70 | 49.2 |
| A9 | 37935 | ˂LD | 0.30 | 46.4 | 125 | 35.0 | 42213 | 6367 | 68.6 | 2.70 | 50.1 |
| A10 | 29902 | ˂LD | 0.70 | 45.00 | 122 | 33.9 | 39734 | 6180 | 66.3 | 3.70 | 48.2 |
| **Average** | **31697** | **-** | **0.48** | **45.1** | **122** | **34.1** | **40417** | **6264** | **66.4** | **3.38** | **48.6** |
| **SD** | **4439** | **-** | **0.15** | **1.38** | **3.92** | **1.14** | **1384** | **128** | **2.21** | **0.56** | **1.25** |
| **Non-mining stations** | | | | | | | | | | |  |
| A11 | 51510 | ˂LD | 0.9 | **31.5** | 135 | 33.9 | 57398 | 1000 | 35.2 | 7.00 | 39.0 |
| A12 | 56199 | ˂LD | 0.9 | 32.8 | 146 | 23.3 | 61813 | 1085 | 37.9 | 8.20 | 41.3 |
| A13 | 64552 | ˂LD | 1.00 | 32.6 | 150 | 23.4 | 63258 | 1013 | 39.8 | 5.80 | 41.8 |
| A14 | 56787 | ˂LD | 0.87 | 30.5 | 142 | 21.8 | 58859 | 1067 | 34.9 | 8.10 | 40.2 |
| A15 | 57211 | ˂LD | 0.88 | 32.2 | 140 | 23.5 | 61023 | 968 | 37.1 | 5.00 | 38.1 |
| **Average** | **57252** | **-** | **0.91** | **31.9** | **142** | **23.0** | **60470** | **1021** | **37.0** | **6.52** | **40.1** |
| **SD** | **4186** | **-** | **0.05** | **0.60** | **6.39** | **0.24** | **2498** | **24.7** | **1.92** | **0.98** | **1.26** |
| **Sampling site** | **Bonikro gold mine site** | | | | | | | | | | |
| **Industrial stations** | **Al** | **As** | **Cd** | **Co** | **Cr** | **Cu** | **Fe** | **Mn** | **Ni** | **Pb** | **Zn** |
| B1 | 34093 | 4.00 | 0.70 | 26.8 | 127 | 19.5 | 39133 | 1031 | 48.3 | 7.10 | 38.5 |
| B2 | 24924 | 15.4 | 0.60 | 18.5 | 80.4 | 18.0 | 30944 | 588 | 16.1 | 8.00 | 32.7 |
| B3 | 17648 | 8.20 | 0.30 | 10.4 | 35.1 | 9.60 | 12098 | 171 | 9.10 | 8.30 | 25.4 |
| B4 | 93330 | 2.00 | 1.30 | 14.8 | 229 | 63.1 | 93823 | 173 | 48.9 | 6.40 | 37.0 |
| B5 | 18900 | 7.30 | 0.30 | 15.5 | 18.6 | 9.10 | 14208 | 555 | 9.10 | 4.70 | 32.3 |
| **Average** | **37779** | **7.38** | **0.64** | **17.2** | **98.0** | **23.9** | **38041** | **504** | **26.3** | **6.90** | **33.2** |
| **SD** | **28377** | **4.39** | **0.37** | **5.45** | **75.6** | **20.1** | **29679** | **319** | **18.4** | **1.29** | **4.57** |
| **Artisanal stations** | | | | | | | | | | | |
| B6 | 46173 | ˂LD | 0.50 | 15.2 | 21.7 | 42.0 | 36307 | 342 | 27.2 | 2.3 | 116 |
| B7 | 63507 | ˂LD | 0.90 | 12.4 | 64.9 | 13.6 | 25447 | 408 | 18.6 | 8.00 | 36.8 |
| B8 | 29959 | ˂LD | 0.50 | 17.6 | 24.4 | 24.3 | 32679 | 286 | 27.1 | 1.70 | 85.5 |
| B9 | 33291 | ˂LD | 0.40 | 8.80 | 26.3 | 12.6 | 27806 | 166 | 13.7 | 10.3 | 39.7 |
| B10 | 7486 | ˂LD | 0.40 | 6.70 | 33.3 | 15.5 | 24070 | 128 | 10.9 | 16.2 | 102 |
| **Average** | **36083** | **-** | **0.54** | **12.1** | **34.1** | **21.6** | **29262** | **266** | **19.5** | **7.70** | **75.9** |
| **SD** | **18531** | **-** | **0.19** | **4.00** | **15.9** | **11.0** | **4581** | **105** | **6.72** | **5.37** | **32.3** |
| **Non-mining stations** | | |  |  |  |  |  |  |  |  |  |
| B11 | 72349 | 113 | 2.70 | 13.0 | 318 | 55.9 | 175213 | 180 | 30.7 | 16.4 | 52.8 |
| B12 | 10326 | 8.00 | 0.50 | 12.6 | 93.9 | 6.80 | 30491 | 649 | 9.00 | 6.40 | 19.9 |
| B13 | 62619 | 7.10 | 1.50 | 16.9 | 439 | 58.1 | 97539 | 109 | 31.6 | 11.4 | 39.1 |
| B14 | 15550 | 6.60 | 0.90 | 31.4 | 357 | 25.7 | 59135 | 735 | 16.0 | 16.9 | 19.1 |
| B15 | 39125 | 33.6 | 0.60 | 16.9 | 301 | 36.0 | 91073 | 397 | 20.7 | 12.3 | 31.0 |
| **Average** | **39993** | **33.6** | **1.24** | **18.2** | **302** | **36.5** | **90691** | **414** | **21.6** | **12.7** | **32.4** |
| **SD** | **27551** | **45.8** | **0.83** | **7.6** | **128** | **21.4** | **54338** | **276** | **9.65** | **4.26** | **14.1** |

*To calculate average values, limits of detection (LD) were used for data < LD.
